# PlantGSAD: a comprehensive gene set annotation database for plant species

**DOI:** 10.1093/nar/gkab794

**Published:** 2021-09-17

**Authors:** Xuelian Ma, Hengyu Yan, Jiaotong Yang, Yue Liu, Zhongqiu Li, Minghao Sheng, Yaxin Cao, Xinyue Yu, Xin Yi, Wenying Xu, Zhen Su

**Affiliations:** State Key Laboratory of Plant Physiology and Biochemistry, College of Biological Sciences, China Agricultural University, Beijing 100193, China; State Key Laboratory of Plant Physiology and Biochemistry, College of Biological Sciences, China Agricultural University, Beijing 100193, China; State Key Laboratory of Plant Physiology and Biochemistry, College of Biological Sciences, China Agricultural University, Beijing 100193, China; State Key Laboratory of Plant Physiology and Biochemistry, College of Biological Sciences, China Agricultural University, Beijing 100193, China; State Key Laboratory of Plant Physiology and Biochemistry, College of Biological Sciences, China Agricultural University, Beijing 100193, China; State Key Laboratory of Plant Physiology and Biochemistry, College of Biological Sciences, China Agricultural University, Beijing 100193, China; State Key Laboratory of Plant Physiology and Biochemistry, College of Biological Sciences, China Agricultural University, Beijing 100193, China; State Key Laboratory of Plant Physiology and Biochemistry, College of Biological Sciences, China Agricultural University, Beijing 100193, China; State Key Laboratory of Plant Physiology and Biochemistry, College of Biological Sciences, China Agricultural University, Beijing 100193, China; State Key Laboratory of Plant Physiology and Biochemistry, College of Biological Sciences, China Agricultural University, Beijing 100193, China; State Key Laboratory of Plant Physiology and Biochemistry, College of Biological Sciences, China Agricultural University, Beijing 100193, China

## Abstract

With the accumulation of massive data sets from high-throughput experiments and the rapid emergence of new types of omics data, gene sets have become more diverse and essential for the refinement of gene annotation at multidimensional levels. Accordingly, we collected and defined 236 007 gene sets across different categories for 44 plant species in the Plant Gene Set Annotation Database (PlantGSAD). These gene sets were divided into nine main categories covering many functional subcategories, such as trait ontology, co-expression modules, chromatin states, and liquid-liquid phase separation. The annotations from the collected gene sets covered all of the genes in the *Brassicaceae* species *Arabidopsis* and *Poaceae* species *Oryza sativa*. Several GSEA tools are implemented in PlantGSAD to improve the efficiency of the analysis, including custom SEA for a flexible strategy based on customized annotations, SEACOMPARE for the cross-comparison of SEA results, and integrated visualization features for ontological analysis that intuitively reflects their parent-child relationships. In summary, PlantGSAD provides numerous gene sets for multiple plant species and highly efficient analysis tools. We believe that PlantGSAD will become a multifunctional analysis platform that can be used to predict and elucidate the functions and mechanisms of genes of interest. PlantGSAD is publicly available at http://systemsbiology.cau.edu.cn/PlantGSEAv2/.

## INTRODUCTION

The continued development and widespread availability of high-throughput techniques allow for the exploration of changes and regulation at a whole genome-wide level under certain conditions. It is important to integrate the large number of data sets generated by high-throughput experiments for gene annotation, especially when working with gene sets. The gene ontology (GO) system is a common functional category widely used in plenty of analysis platforms and tools, such as DAVID (1,2) and agriGO (3,4). Due to the limitation of GO gene sets, several platforms, such as MSigDB (5), WebGestalt (6) and PlantGSEA (7), have been designed to assign genes to categories based on Kyoto Encyclopedia of Genes and Genomes (KEGG) metabolic processes, gene families, and some curated gene sets. All of these gene sets can be used to compute the overlap with a query gene list for the biological interpretation using gene set enrichment analysis (GSEA) (8).

The biological knowledge associated with gene sets is gradually extending beyond pre-existing gene set categories, with multiple diverse data sets emerging and new functional categories being introduced. For example, co-expression modules (CoMs) (9) are a new functional category that has been added into some platforms and databases, including EviNet (10), WebGestalt 2017 (6), ccNET (11) and MCENet (12). In addition, various forms of epigenetic regulation, such as DNA methylation, histone modifications, and histone variants, have been widely implicated in the control of complex biological activities (13). Specific combinations of multiple epigenetic marks at the whole genome level can be defined as chromatin states, which play a meaningful role in various biological processes. Recently, there have also been significant advances in research on liquid-liquid phase separation (LLPS), single cell RNA-seq and RNA binding proteins in biological systems, leading to new data types. It has been reported that LLPS is associated with chromatin compartmentalization in transcriptional regulation (19–21). At the same time, plant research has extended into three-dimensional (3D) genomics analysis (14) and 3D genomic data from various plant species have been published using Hi-C and ChIA-PET techniques (15–18). However, 3D-related categories for gene sets associated with Hi-C and LLPS are seldom present in databases and platforms for search and analysis.

Although some resources with gene set annotations and analyses are available, including PAGED (22) and GeneSetDB (23), they mainly focused on the disease-related gene sets in human and typical gene sets from model species. In addition, both PAGED (22) and GeneSetDB (23) were published in 2012 and lacked novel data types such as chromatin state-based gene sets, LLPS gene sets, and single-cell RNA-seq cluster gene sets. For plants, the functional genome database of some species covers the common types of gene sets. For example, The Arabidopsis Information Resource (TAIR) (24) database offers GO and KEGG categories for *Arabidopsis*. However, there is no existing database that broadly integrates gene sets across different categories for plant species or that provides gene annotations at multidimensional levels for plant communities. Therefore, it is necessary to expand the number of data types and gene sets for a wider range of plant species and to logically organize the gene set categories.

To meet the growing demands of researchers and users, we built the Plant Gene Set Annotation Database (PlantGSAD) and uploaded enough novel categories of gene sets from plant species, including CoMs, chromatin states, and LLPS. Currently, PlantGSAD contains 236 007 gene sets across nine main functional categories for 44 plant species. All of these gene sets can be browsed and specific detailed knowledge of gene sets can be searched for. In addition, GSEA analysis is available in the background for the collected gene sets to produce comprehensive output report and graphical visualization for each gene set category. Other useful tools in PlantGSAD include customized singular enrichment analysis (SEA), SEACOMPARE (the cross comparison of SEA results), dot plot drawer and ID conversion. We believe that PlantGSAD is beneficial for gene function analysis and as a reference platform for plant species. It is freely available at http://systemsbiology.cau.edu.cn/PlantGSEAv2.

## MATERIALS AND METHODS

### Data sources and integration

We collected gene sets across different types of data from various resources, and organized them using a curation workflow. The data sources used for gene sets are listed in Table 1 and Supplementary Table S1.

**Table 1.** Total numbers and sources of the categorized gene sets for comprehensively annotated species

Category			No. of supported organisms	No. of total gene sets	No. of covered genes	Main data source
G1	GO	Gene Ontology	44	105 339	878 035	AgriGOv2 (3)/TAIR (24)/Phytozome (25)
G2	PO	Plant Ontology	3	5561	44 500	Planteome (26)/TAIR (24)
	TO	Plant Trait Ontology	4	3319	1066	RiceData /Planteome (26)
	PECO	Plant Experimental Conditions Ontology	1	564	20 392	Planteome (26)
G3	Cyc	PlantCyc	33	56 606	210 169	PlantCyc (28)
	KEGG	KEGG	39	16 494	456 730	KEGG (27)/Phytozome (25)
	Map	MapMan	14	17 426	507 408	MapMan (29)
G4	TR	Transcription Regulators/Factors	41	2851	84 041	PlantTFDBv4.0 (68)
	CAZy	Carbohydrate-Active Enzymes	9	842	10 754	CAZy database (69)
	PK	Protein Kinase	8	574	9714	MCENet (12)/ccNET(11)/iTAK (70)
	Ub	Ubiquitins	15	529	17 373	iUUCDv2.0 (30)/PlantsUPS (71)
	P450	Cytochrome P450	8	897	2555	MCENet (12) /ccNET(11) /SorghumFDB (72)
	EAR	EAR motif	35	1216	9223	PlantEAR (37)
	LIP	Lipid metabolism Enzymes	2	359	1873	ARALIP website (73)/SFGD (74)
	Caf	Chromatin associated factor	2	166	1078	ChromDB (75)
	OTH	Other gene family based gene sets	3	703	5782	TAIR/SorghumFDB (72)
G5	Sta	Chromatin states	5	159	205 647	PCSD (31)
	ERG	Epigenetic mark related genes	4	288	170 572	PCSD (31)
	CRG	Chromatin associated factor related genes	3	44	46 944	PCSD (31)
	HiC	Hi-C identified genes	2	4	4031	2 literatures
G6	TFT	Transcription Factor Targets	2	760	64 810	Plant Cistrome Database (38)/AGRIS (76)/
	MIR	MicroRNA Targets	9	4694	25 614	AraPath (77)/PNRD (78)
G7	CoM	Co-expression gene module	10	14 220	82 020	ATTED-II (79) /ccNET (11) /SorghumFDB (72) /MCENet (12)
G8	LIT	Literature/Reference gene sets	16	1950	110 503	458 literatures
G9	ScR	Single cell RNA-seq identified gene sets	4	337	32 574	7 literatures
	LLPS	Liquid-liquid phase separation related genes	31	93	194 564	DrLLPS (80)/PSPredictor (81)
	Rbp	RNA binding protein	1	12	2766	4 literatures

The ontology gene sets included gene ontology (GO), plant ontology (PO), and trait ontology (TO); these were downloaded from related annotation databases, such as agriGOv2 (3), Phytozome (25), TAIR (24), Planteome (26) and RiceData (http://www.ricedata.cn). The PO and TO gene sets were re-computed to cover every term (including the parent terms) and organized as formatted annotation files. We also collected the gene sets from KEGG (27), PlantCyc (28) and MapMan (29), and grouped them as pathway gene sets.

With regard to the gene family-based gene sets, the gene families were collected from genomics platforms (e.g. MCENet (12) for maize and ccNET (11) for cotton), and specific gene family databases (e.g. PlantTFDBv4.0 (68) for transcription factor families and iUUCDv2.0 (30) for ubiquitin proteasome system-related gene families). The collected data were manually curated to remove redundancy and conflicts.

We also integrated the gene sets related to transcription regulation. The data sets and genome-wide annotation in PCSD database (31) were organized into chromatin state (Sta) gene set, epigenetic mark related gene set (ERG), and chromatin associated factor related gene set (CRG). We grouped them together with the Hi-C gene set (HiC) to define a new category: chromatin states based gene sets. We also integrated information related to transcription factor targets and microRNA targets from multiple data sources, such as the Plant Cistrome Database (38), AGRIS (76) and PNRD (78), to define another category: target gene sets.

We defined a CoM gene set based on co-expression gene networks within the public resources. For the species such as maize and cotton, the CoM gene sets were directly downloaded from MCENet (12) and ccNET (11). For the species such as *Arabidopsis* and rice, we collected the co-expression gene network built by ATTED-II (79) and RiceFREDN (32) and identified the functional modules by CFinder (33) with standard parameters. In addition, we manually collected reference gene sets from published literatures, which then underwent curation and organization. We also grouped some gene sets related to novel functional categories, such as single-cell RNA sequencing (scRNA-seq), liquid–liquid phase separation (LLPS), and RNA-binding proteins (RBPs). These gene sets were manually collected from specific web servers and literatures (listed in Table 1 and Supplementary Table S2).

For some species, the collected gene sets might be got from different genome versions. We converted them to the unified ID system for the same species to allow curation, browse and analysis (Supplementary Table S3).

### Functional category enrichment analysis

In analysis page of PlantGSAD, users can submit a list of genes of interest for the enrichment analysis, and choose suitable categories from G1 to G9. Statistical tests and multiple test correction methods are provided. Three statistical tests are available for selection: hypergeometric test, Fisher's exact test and chi-square test. The statistical formula for Fisher's exact test, which is the default test, is presented in Equation (1):

(1) $$\begin{equation*}{\rm P}=\frac{(_{k}^{n})(_{K-k}^{N-n})}{(_{K}^{N})}\end{equation*}$$

where *N* is the total number of genes in an organism or in the user-provided background, *n* is the number of genes in the query list, *K* is the total number of genes in one gene set and *k* is the number of overlapping genes.

We provide six multiple test correction methods: Yekutieli, Bonferroni, Hochberg, Benjamini-Hochberg (BH), Hommel and Holm (34). Adjusted P-values are obtained after performing correction for the false discovery rate (FDR), with the significantly enriched gene sets for each gene set category displayed using a cut-off selection.

### SEACOMPARE and custom SEA

Users can compare two or more SEA results using the SEACOMPARE tool, with a heatmap showing the common or unique significant gene sets. The color indicates the P-value or FDR. The custom SEA is independent of species and shares the same principle as functional category enrichment analysis. To use this custom tool, users need to prepare background datasets consisting of genes and their related gene sets. If the parent-child relationships between gene sets are submitted, the genes in the child gene sets belong to the parent gene sets by default during the enrichment computation. DAG (direct acyclic graph) graphs are also created as part of the results.

### Database implementation

PlantGSAD was constructed on a standard LAMP (Linux + Apache + MySQL + PHP/Python) system. The data sets are stored in MySQL (www.mysql.com) and formatted text files, analysis codes were compiled using Python (www.python.org), and the web interface was built using PHP scripts (www.php.net) on Red Hat Linux powered by an Apache server (www.apache.org). No software or plug-ins are needed because the PlantGSAD tools are web-based. Users are free to access the database and there are no login requirements.

## RESULTS

### Gene set annotation and organization

**Figure 1.**
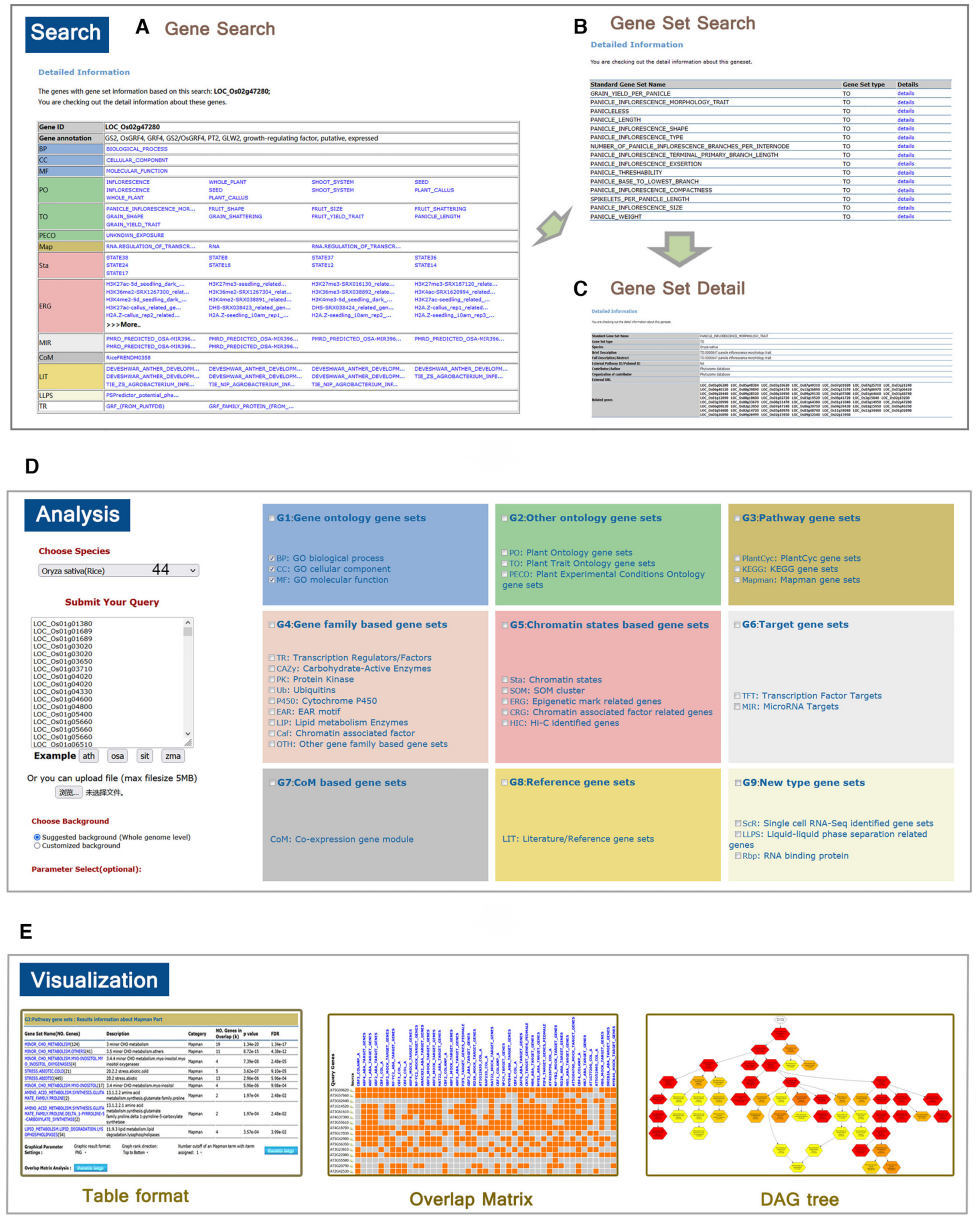
Overview of PlantGSAD functions. (**A**) Example of gene search results in PlantGSAD. In the first column, the background color represents the gene set category. (**B**) Gene set search results in PlantGSAD for the TO category in rice. (**C**) Detailed information on the gene set PANICLE_INFLORESCENCE_MORPHOLOGY_TRAIT. (**D**) GSEA in PlantGSAD. The query gene list for 44 plant species can be input for enrichment analysis (left). Individual gene set categories can be selected (right). (**E**) Enrichment analysis results can be visualized using table, overlap matrix, and DAG tree.

The accumulation of multidimensional omics data and the emergence of new types of data prompted us to collect a large volume of data for various species. The resulting PlantGSAD is capable of annotating gene sets for multiple assay types, including omics data sets generated from high-throughput transcriptomics and epigenomics experiments. PlantGSAD supports 44 species across nine functional categories, including GO (G1), other ontology (G2), pathway (G3), gene family-based (G4), chromatin states-based (G5), target (G6), CoM-based (G7), reference (G8), and new type (G9) gene sets (Figure 1D and Table 1).

The number of defined gene sets is currently 236 007, covering a range of data types from public databases and manually collected literature data (Supplementary Table S2). In addition to GO, the ontology systems have been expanded to include PO, TO, and Plant Experimental Conditions Ontology (PECO), which allows the further annotation of gene sets (26). TO gene sets contain information on the agronomic traits of crops. The GO and other ontology categories are particularly useful for geneticists, biologists, and breeders when investigating the potential molecular mechanisms associated with target genes (35).

For the pathway gene sets, we integrated the KEGG, PlantCyc and MapMan gene sets. MapMan was specifically designed to cover plant-specific pathways and processes (36). We also introduced a number of gene family gene sets, including the EAR motif-related gene family and chromatin associated factors. EAR motif-containing proteins, which are highly conserved transcription repressors across a diverse range of plant species, can interact with co-repressors, affecting the structure of chromatin via histone modifications, thus repressing gene transcription and playing a role in hormone signal transduction, stress responses, and development (37). For this reason, the EAR subcategory was classified as a pivotal gene family in PlantGSAD.

Co-expression networks have emerged as an efficient way to decipher the potential function of genes. We obtained 14 220 CoMs across 10 organisms for PlantGSAD as a functional category. Every CoM was defined as a gene set. CoMs provide basic annotated information to describe the complex roles of gene sets at the transcriptomic level. The complex signaling networks associated with transcriptional changes during developmental stages and responding to environmental cues often involve many related transcription factors (TFs). Thus, transcriptional factor targets (TFTs) were classified as a subtype of target gene sets in PlantGSAD to provide a framework for understanding the regulation mechanisms during plant development processes and stress responses. A large number of gene sets for this functional subcategory were collected for PlantGSAD. For example, we integrated 608 *Arabidopsis* gene sets mainly acquired from DAP-seq (38) and 115 maize TFT data sets primarily obtained from ChIP-seq (39). These TFT data sets in PlantGSAD can be used to investigate query genes involved in complex transcription regulatory networks, providing a valuable genome annotation resource for identifying potential key TFs that transcriptionally regulate the biological processes involving the query genes.

Epigenomic data represents vital information in investigating the dynamic role of chromatin states in gene regulation, notably the linear partitioned genome segments with various epigenetic features localized in unannotated regions (31,40). Thus, we added chromatin state-based gene sets as an essential functional category in PlantGSAD. The increase in the functional categories related to chromatin states is beneficial for increasing the biological understanding of various epichromatin states during developmental processes and in response to environmental cues, and their subsequent differences in transcriptional regulation. Corresponding epigenetic marks, such as DNA methylation, post-translational histone modifications, and chromatin associated factors, are highly dynamic, with the changes in the chromatin states representing distinct epigenetic and spatial features of various transcriptional activities. The chromatin architectural capture technique Hi-C has been widely employed in the characterization of chromatin architecture in various plant and animal model species, leading to the definition of various chromatin architectural features, such as the chromosome territory, compartment A/B, topological associated domains (TADs), and chromatin loops (41). Hence, chromatin states based gene sets were added as a functional category in PlantGSAD, consisting of four subgroups: Sta (defined chromatin states) from the PCSD database, ERG (epigenetic mark related), CRG (chromatin associated factor related), and HiC (Hi-C technology based) gene sets. Every defined chromatin state in a specific species touches on some preferential epigenetic marks in preferentially located regions. The same chromatin state has genes with similar epigenetic marks in similar regions of the genes, which might jointly participate in the transcription regulation of cellular activities. In addition, the information about genome-wide chromosome conformation capture can help to identify genes related to the assembly and conformational changes of chromatin.

Reference gene sets were also curated, organized, and integrated from public research information, especially up to 1636 manually collected reference gene sets for *Arabidopsis*. We also introduced more novel functional categories to PlantGSAD to better serve the needs of biologists from different research areas. Single-cell RNA sequencing (scRNA-seq) has been extensively employed to study cell-specific gene expression in animals and plants. The genes from distinct subpopulations and rare cell types of plants, such as putative quiescent center cells, have been included for the gene set annotation. As a critical component of chromatin compartmentalization, LLPS drives the formation of miscellaneous membrane-less compartments in cells and is involved in 3D chromatin organization and transcriptional regulation. Due to the fact that LLPS is associated with chromatin compartmentalization in transcriptional regulation, we integrated the LLPS-related proteins and included related gene sets in a functional sub-category. In the process of post-transcriptional regulation, RBPs are indispensable chaperones that naturally bind to RNA via one or multiple globular RNA-binding domains, changing the function and/or fate of the bound RNAs (42–46). Because RBPs are critical components in adjusting global cellular transcript levels via the binding to and potential regulation of the transcripts, we collected these proteins as a novel gene set type.

PlantGSAD contains cereal crops, economic crops, and medicinal plants, etc. In particular, it includes vegetable and oilseed crops in the *Brassicaceae* family and major cereal crops within the *Poaceae* (grass) family. In PlantGSAD, the annotations from collected gene sets cover all of the genes in the *Brassicaceae* species *Arabidopsis* and the *Poaceae* species *Oryza Sativa* (rice), while the annotation rate for maize (*Zea Mays*) genes has reached over 99 percent. In addition, soybean (*Glycine max* L.) in *Fabaceae* produces high-quality oil from seeds, so functional annotations of the genes involved in the soybean acyl-lipid pathway have been collected and grouped into the lipid metabolism enzymes (LIP) subcategory. Medicinal plants have also attracted significant research attention (for example, *Catharanthus roseus* has been shown to produce anti-cancer agents), thus we collected CoM data sets of *C. roseus* from CroFDB database (47) for use in PlantGSAD.

The large number and the wide range of gene sets from plant species in PlantGSAD will significantly enhance the biological interpretation of genes of interest and cover a large range of unknown genes.

### Gene set query and access

All of the gene sets from the 44 plant species can be easily accessed and checked individually using the free selection of categories and species on the browse page of PlantGSAD (Figure 1). The name, type, species, and annotations for these gene sets are presented and the name of the gene set links to a details page for more information (Figure 1A). Rice gene LOC_Os02g47280 (*OsGRF4*, growth-regulating factor 4) is used as an example for the search function in the PlantGSAD (Figure 1A). The search tools help users to conveniently retrieve the detailed information of collected gene sets and their constituent genes, and up to 30 genes can be searched for at the same time. A keyword-based search is provided for the gene sets (Figure 1B, C). The detailed annotations for the gene sets or individual genes serve as important selection criteria when choosing candidates for follow-up studies.

Furthermore, GSEA analysis is available in the background of all gene sets in PlantGSAD, so the query gene list can be annotated using the enriched gene sets (Figure 1D). The direct visualization of the GSEA output report is convenient for analyzing the relevant biological knowledge (Figure 1E). PlantGSAD is also capable of analyzing query lists of genes from one or more functional categories, and the GSEA results are presented clearly in a series of tables. The database provides a summary head in the result page highlighting the significant results in each category and presenting a link to the corresponding analysis table. For ontological analyses (e.g. PO and TO), we integrated visualization features (e.g. DAG) to intuitively reflect the parent-child relationships. DAGs highlight the essential and meaningful terms from the raw and partially redundant enrichment results, allowing researchers to better understand the nature of the causal relationships involved. Furthermore, the significance levels of the terms in the DAGs are directly indicated using a color range from light yellow to dark red.

### Additional assistant tools for analysis

Several tools can be implemented into PlantGSAD to improve the efficiency of the analysis. Custom SEA tools have been designed to adapt to individual user-defined data ranging from specific plants to animals or other organisms, thus providing a flexible strategy based on a customized background and the optional relationship of terms. If users provided a query list and customized background, they could gain GSEA results for their area of interest. DAG graphs determining the relationship between terms are also produced if a reference file for relationships is provided. Cross-comparison is also vital for interpreting the analysis results obtained from experiments involving multiple samples, such as time-series experiments (Figure 3). Users can submit three formats for SEACOMPARE analysis, including customized datasets, result tables from GSEA analyses, and multiple session/job IDs. All of these formats contain multiple numeric values from separate experiments. In addition, an ID conversion tool is available for some species in PlantGSAD.

### Application of GSEA with rice OsGRF4 binding genes

**Figure 2.**
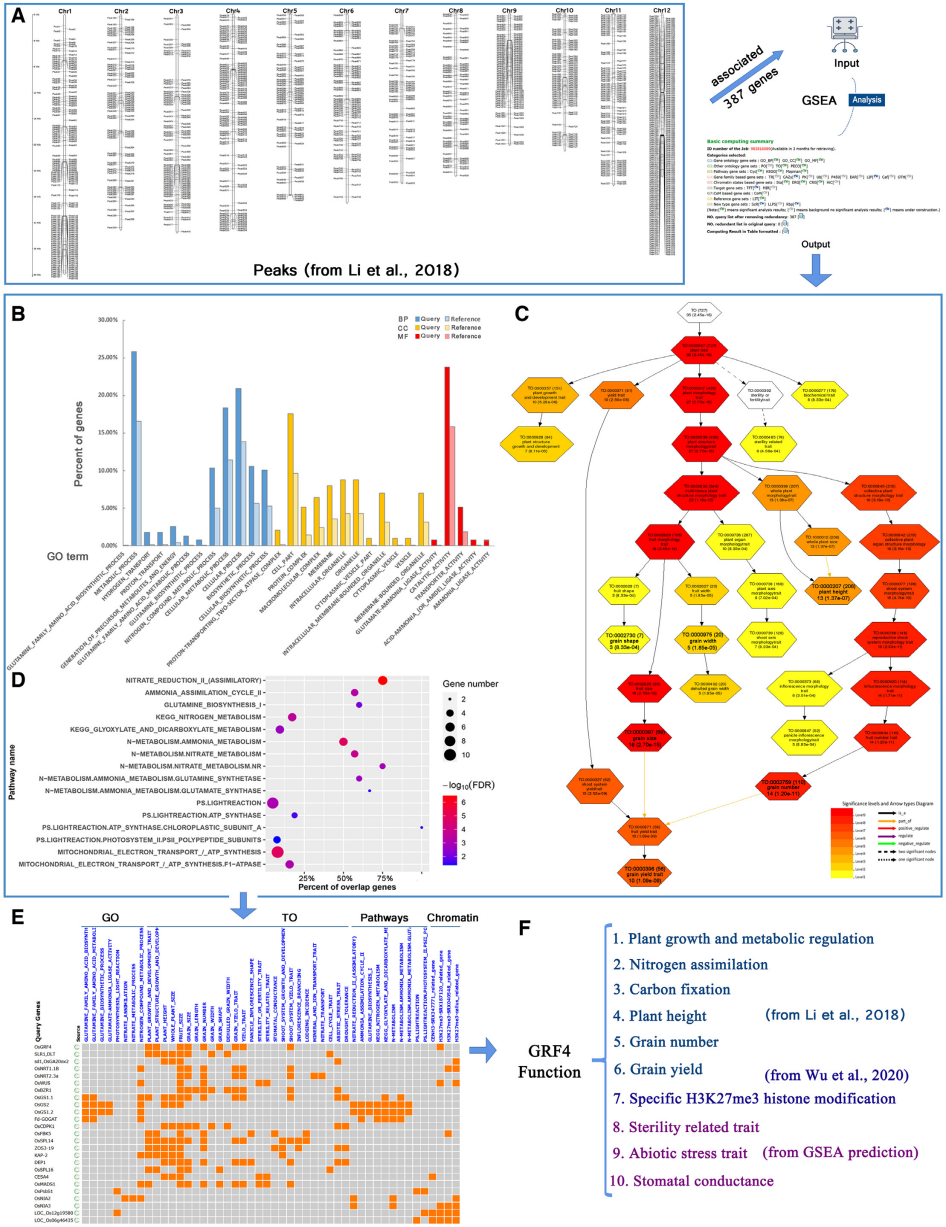
Gene sets enrichment analysis of OsGRF4 binding genes. (**A**) Distribution of OsGRF4 binding peaks in the 12 rice chromosomes. (**B**) Enriched GO terms from the GSEA results. (**C**) Enriched pathways from the GSEA results. (**D**) DAG for the enriched TO terms with a cutoff FDR of 0.001. (**E**) Overlap matrix for GO-, TO-, pathway- and chromatin state-related gene sets. (**F**) Overview of GRF4 function.

In this section, we present an example of the use of PlantGSAD for gene set enrichment analysis (GSEA) of a gene list of interest to illustrate the features of the multiple functional categories in the database. Rice GROWTH-REGULATING FACTOR 4 (OsGRF4) is a positive transcriptional regulator of multiple nitrogen-metabolism genes and coordinates carbon metabolism and growth (48). We utilized a group of target genes associated with OsGRF4 binding peaks identified in a ChIP-seq experiment (48) as a query gene list for GSEA. The OsGRF4 binding peaks cover 387 genes spreading all over the 12 chromosomes of rice (Figure 2A). GSEA was initially conducted with these 387 genes. Gene sets from the GO, TO, PECO, metabolic pathways, and chromatin states categories were significantly enriched.

In the GO (G1) category, there were some enriched biological processes related to key nitrogen-assimilation enzymes (Figure 2B), such as nitrate assimilation (related to assimilation), glutamine family amino acid biosynthetic process, and glutamine biosynthetic process (related to assimilation), which were confirmed by the enrichment analysis results derived from agriGOv2 (Supplementary Table S4). In the TO category, a number of agronomic traits were enriched, especially plant growth and development trait, grain number and grain yield trait (Figure 2C). The pathways such as nitrate reduction II (assimilatory), Nitrogen metabolism, PS.lightreaction were also enriched (Figure 2D). In particular, the enrichment of PS.lightreaction indicated that these query genes had effects on photosynthesis and carbon-assimilation. The GO and TO gene set enrichment analysis results indicated that OsGRF4 regulates downstream genes and coordinates both nitrogen and carbon assimilation, which is consistent with previous research that has reported that OsGRF4 is a positive regulator of plant carbon and nitrogen metabolism, photosynthesis, maintaining the C:N ratio, and promoting plant growth and development (48).

Interestingly, in the analysis of chromatin states based gene sets, we found that CENH3-related and H3K27me3-related genes were significantly enriched (Supplementary Table S4). This may be related to the recently reported enhancement of the sustainable green revolution yield via nitrogen-responsive chromatin modulation in rice (49). NITROGEN-MEDIATED TILLER GROWTH RESPONSE 5 (NGR5) interacts with a component of the polycomb repressive complex 2 (PRC2) and alters the H3K27me3 pattern response to changes in nitrogen availability. There exists a genetic interaction between GRF4 and NGR5, and the accumulation of these proteins can improve the yield and nitrogen-use efficiency of main rice varieties under reduced nitrogen fertilizer loadings (49). Thus, our enrichment analysis results for chromatin states-based gene sets suggest that OsGRF4 might regulate downstream genes via the NGR5-dependent recruitment of PRC2 and the reprogramming of H3K27me3 methylation.

We then conducted overlap matrix analysis for the enriched gene sets to isolate the related essential genes (Figure 2E). These included *SLR1* (50,51)*, sd1* (52,53)*, OsNRT1.1B* (54)*, OsNRT2.3a* (55)*, OsWUS, OsBZR1* (56)*, OsGS1.1, OsGS1.2, OsGS2* (57) and *OsCDPK1* (58,59). These genes physically interact with the rice GRF4 and DELLA protein SLR1 to modulate plant growth and metabolic co-regulation. Moreover, *OsNRT1.1B* and *OsNRT2.3a* encode uptake transporters, and *sd1* and *OsBZR1* conferred semi-dwarf leads to GRV resistance to yield-reducing lodging (i.e. the flattening of plants by the wind and rain).

Finally, we summarized known and unknown GRF4 functions from published papers (Figure 2F). Using GSEA in PlantGSAD and follow-up prediction, it was shown that GRF4 not only promotes nitrogen assimilation, carbon fixation, plant height, and grain yield and growth, but also takes part in the response to abiotic stress and other predicted biological processes. In addition, the target genes for OsGRF4 are possibly involved in H3K27me3 histone modification.

The above functional analysis results demonstrated that PlantGSAD is a powerful online database with a large gene set coverage and multiple visual graph tools for the enrichment analysis of functional categories.

### Application of SEACOMPARE tool

**Figure 3.**
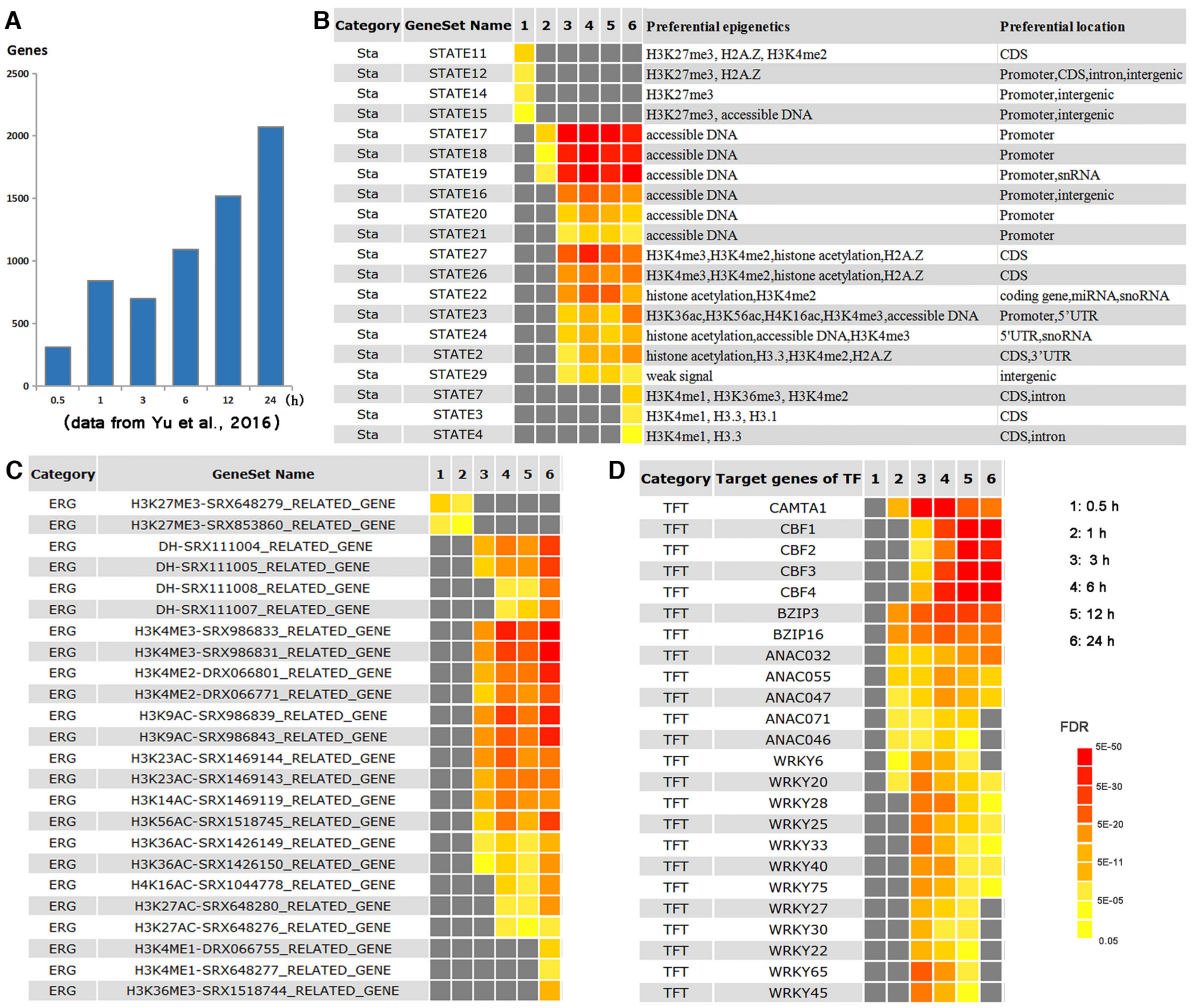
SEACOMPARE analysis of GSEA results for the up-regulated gene list after a 0.5-, 1-, 3-, 6-, 12- or 24-h of cold treatment in *Arabidopsis thaliana*. (**A**) Number of up-regulated genes after a 0.5-, 1-, 3-, 6-, 12- or 24-h of cold treatment in *A. thaliana*. (**B-D**) SEACOMPARE analysis of GSEA results for chromatin states (Sta), epigenetic mark related (ERG), and transcription factor targets (TFT) gene sets. The columns present, from left to right, the subcategory, gene set name, and a heatmap (columns 3–9). The Sta subcategory has two additional annotated columns for preferential epigenetics (right). The colored blocks in the heatmap represent the level of significance for each term. Moving from yellow to red indicates a movement from low to high significance; grey indicates not significant.

In PlantGSAD, several singular enrichment analysis (SEA) results from different samples can be compared using the SEACOMPARE tools. We thus investigated dynamic changes in chromatin states and the epigenetic and transcriptional regulators of up-regulated genes under cold treatment with time series in the model plant *Arabidopsis thaliana* (60). The numbers of gene associated with cold treatment of 0.5, 1, 3, 6, 12 and 24 h were 302, 594, 768, 1055, 1214 and 1205, respectively (Figure 3A and Supplementary Table S5). Using enrichment analysis of chromatin state and TFT gene sets, the temporally dynamic response to cold stress at the epigenetic (Figure 3B, C) and transcriptional level (Figure 3D) was determined.

For convenience, we describe here only a selection of the gene sets from the chromatin, ERG and TFT categories (complete SEA comparison results are presented in Supplementary Table S6). Intriguingly, the STATES 11, 12, 14 and 15, mainly corresponding to the repressed epigenetic mark H3K27me3, were enriched for the up-regulated genes at the early stage of treatment (0.5 h). Over time, however, STATES 17, 18, and 19 became enriched (1-24 h), followed by STATES 16, 20 and 21 (i.e. 3, 6, 12 and 24 h). The SEACOMPARE results indicate that the genes modified with the repressed epigenetic mark H3K27me3 tend to function at the beginning, followed by genes with the accessible regions. In the ERG category, the STATES 27, 26 and 22 conformed to genes with H3K4 tri-methylation or di-methylation modifications. STATES 22-24 and 2 are mainly associated with histone acetylation. After 24 h of cold treatment, the STATES 7 and 3-4 were significantly enriched; these were associated with H3K4me1 and H3K36me3 modification (Figure 3B, C).

The SEACOMPARE results for the TFT subcategory showed that, among the genes induced by 1 h of cold treatment, there was an enrichment in the binding sites for CALMODULIN-BINDING TRANSCRIPTIONAL ACTIVATOR 1 (CAMTA1). Previous studies (61,62) have suggested that CAMTA1 and CAMTA2 work in concert with CAMTA3 at low temperature (4°C) to increase the transcript levels of CRT/DRE BINDING FACTOR (CBF) genes after 2 h. The key regulatory CBF pathway confers freezing tolerance in Arabidopsis and other plants (63,64). Our SEACOMPARE results showed that the targets of CBF1, CBF2 and CBF3 were enriched after from 3 h of cold treatment (Figure 3D). Interestingly, the enrichment of target genes of CBFs reached a maximum level at ∼24 h. In addition, several other transcription factors, such as BZIPs, ANACs, and WRKYs may contribute to genes induced at 0.5-24 h cold treatment to enhance tolerance to freezing. For example, WRKY6 has been reported to positively regulate freezing tolerance (65).

Overall, using SEACOMPARE tool on of chromatin states, ERGs and TFTs, the temporally dynamic regulation changes of genes and their progressive waves of transcriptional responses during cold stress were able to be monitored. Thus, this tool allows users to quickly and efficiently decipher the dynamic changes in regulation and biological processes during different developmental stages and in relation to stress responses over time.

## DISCUSSION

The emergence of numerous novel data types and high-throughput data sets for plant species over recent years has raised the urgent need to comprehensively integrate and effectively process annotated gene sets. The gene set annotation databases for human and model species PAGED (22) and GeneSetDB (23) were published in 2012, and the platforms DAVID (2,66), MSigDB (5), and WebGestalt (6) offer some functional categories for gene sets. However, they primarily focus on the gene-set annotation for human and model species, and there are a very limited number of plant species in these platforms. Therefore, we created PlantGSAD, a comprehensive gene set annotation database for multiple plant species. PlantGSAD integrates 236 007 gene sets across nine different categories for 44 plant species, consisting primarily of cereal crops, economic crops, and medicinal plants. Compared with other platforms, PlantGSAD covers a more comprehensive range of categories, including chromatin states based gene sets (G5) and new type gene sets (G9) such as LLPS and scRNA-seq gene sets. In addition, PO, TO and other plant-related categories are available in PlantGSAD, increasing the annotating possibilities.

In PlantGSAD, we provide the basic GSEA, which is a powerful functional analysis method for interpreting the biological meaning of a group of genes. We originally developed the web-based PlantGSEA (7) in 2013, which has a user-friendly interface and a performance-efficient framework. We adapted the original GSEA tools from PlantGSEA for use in PlantGSAD, while several new tools were also developed to improve the analytical efficiency, including DAG for ontology categories, SEACOMPARE, and custom SEA. The DAGs intuitively reflect the parent-child relationships in ontology analysis, while SEACOMPARE was developed to allow the cross-comparison of SEA results. The present paper presented an example of this tool being used to investigate the dynamic changes in chromatin states and transcription regulation from time-series transcriptomic data during cold stress in *Arabidopsis*. The custom SEA tool was established to process individual user-defined data on a customized background and the optional relationship of gene sets.

It is important to note that PlantGSAD has some limitations and room for improvement. In particular, there are plans to add more gene sets, data types, species, visualizations, and tools. We will increase the types of gene sets for the supported species from the published literatures and new databases. For example, Li *et al.* (67) recently reported a high-resolution cell atlas for the xylem in *Populus* using scRNA-seq, which has already been added to our database as a key ScR gene set for *Populus*. We plan to routinely update the genome version of the available plant species around every six months. We also plan to add gene sets for new plant species in PlantGSAD, and this process may take longer time due to the accumulation of various types of gene sets from different resources. Furthermore, we have set up the custom SEA tool to satisfy the urgent requirements for the data types or the species that were not included in the existing data sets.

In PlantGSAD, the large number and the wide variety of gene sets from multiple species, together with the high-efficiency analysis tools and visualization features, greatly assist the biological interpretation of genes of interest in plants. We believe that the multidimensional plant gene annotations in PlantGSAD can be used to conveniently interpret the biological meaning of a gene via its connectivity and relationships, thus it represents a promising multifunctional platform for the functional analysis of plant genes.

## Supplementary Material

gkab794_Supplemental_Files
