# Supplementary material for: PlantGSAD: a comprehensive gene set annotation database for plant species
Source: Nucleic Acids Res. 2021 Sep 17;50(D1):D1456–67. doi: 10.1093/nar/gkab794 (PMC8728169; doi:10.1093/nar/gkab794)
Supplement: gkab794_Supplemental_Files [file gkab794_supplemental_files.zip › SUPPLEMENTARY INFORMATION.docx]

**SUPPLEMENTARY INFORMATION**

**Supplementary Table 1.** Sources of the categorized gene sets for comprehensively annotated species.

**Supplementary Table 2.** Number of categorized gene sets for comprehensively annotated species.

**Supplementary Table 3.** Species information for comprehensively annotated species.

**Supplementary Table 4**. GSEA results for OsGRF4 binding genes

**Supplementary Table 5**. Up-regulated gene list after 0.5-, 1-, 3-, 6-, 12-, or 24-h of cold treatment in *A. thaliana*.

**Supplementary Table 6**. SEACOMPARE results for up-regulated gene lists after the cold treatment in *Arabidopsis thaliana*.
